# Successful ablation of atrial fibrillation in a patient with a highly calcified septum after an atrial septal defect operation

**DOI:** 10.1002/joa3.12846

**Published:** 2023-04-01

**Authors:** Hitoshi Mori, Wataru Sasaki, Taisuke Nabeshima, Kenta Tsutsui, Ritsushi Kato

**Affiliations:** ^1^ Department of Cardiology Saitama Medical University International Medical Center Saitama Japan; ^2^ Department of Pediatric Cardiology Saitama Medical University International Medical Center Saitama Japan

**Keywords:** atrial fibrillation, atrial septal defect, intracardiac echocardiography, patch closure, trans septal puncture

As a result of the improvement in surgical treatment over the past few decades, the life expectancy of patients with an atrial septal defect (ASD) has been substantially extended. Consequently, as they age, the incidence of atrial arrhythmias including atrial fibrillation (AF) increases. Catheter ablation (CA) is an effective treatment for AF, which relieves the symptoms and reduces heart failure hospitalizations. A transseptal puncture (TSP) into the left atrium (LA) after an ASD operation is sometimes challenging due to prior operations. After a surgical repair with a septal stitch, the TSP passes through the repaired septum. Likewise, in patients with a pericardial or Dacron patch, the TSP is performed directly through the patch or the surrounding native interatrial septum.^1^ However, evidence is scarce in cases after an ASD operation, who have a narrow residual native interatrial septum with a highly calcified repaired patch, which precludes a direct puncture.

A 72‐year‐old woman with a previously repaired ASD performed 52 years prior, and of which the detailed information of the surgery was missing, was referred to our hospital for CA of symptomatic, drug resistant AF. The LA diameter was 48 mm, but no other abnormalities such as a septal calcification were observed on transthoracic echocardiography (TTE).

Prior to the CA, computed tomography (CT) was performed to assess the cardiac anatomy. We observed that the repaired atrial septum was highly calcified, and the residual native septum length was <1 cm (Figure 1A), suggesting difficulty for a direct puncture through the calcified part of the septum, and thus we might need to pass through the narrow native septum.

**FIGURE 1:**
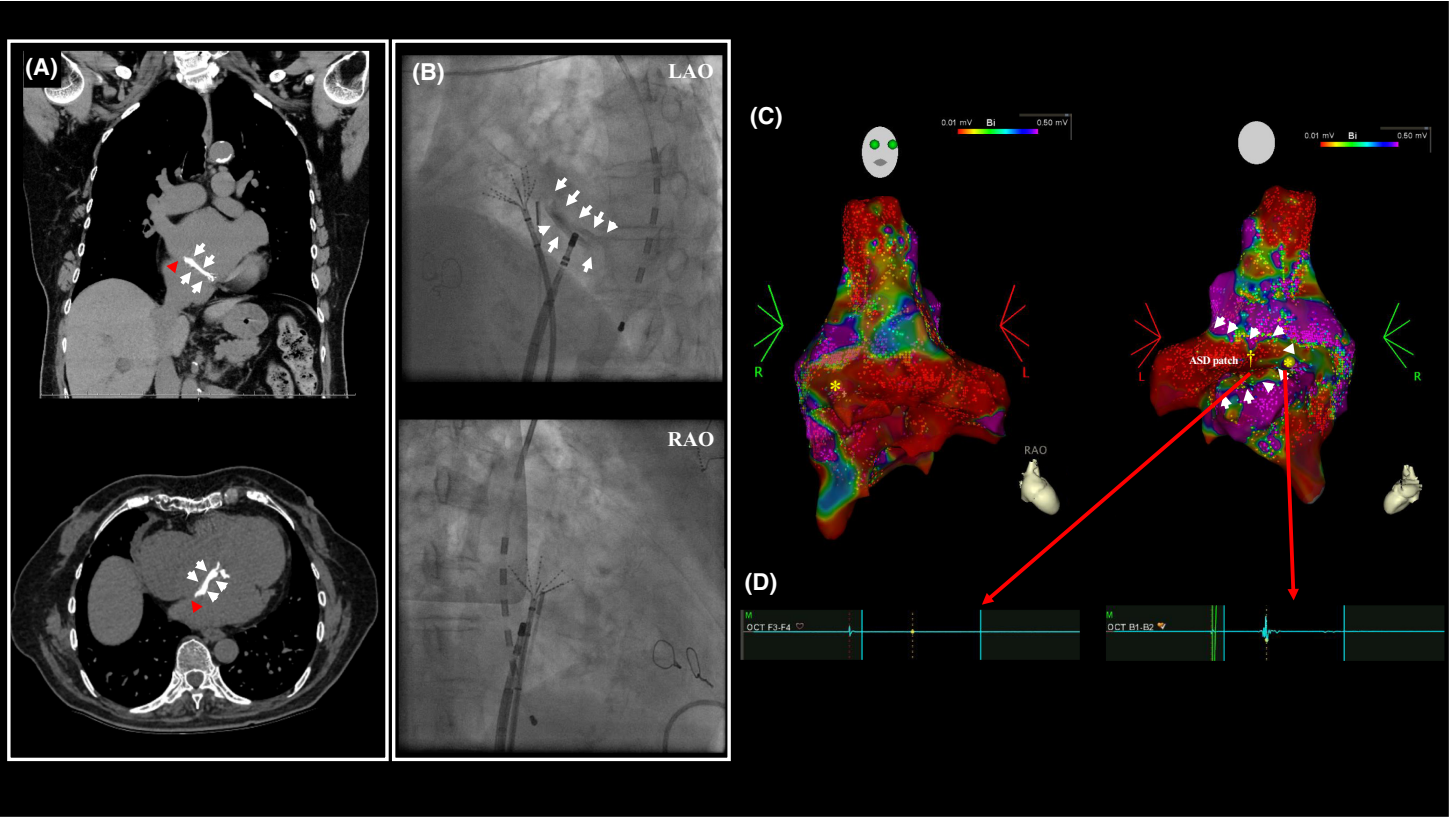
(A) A computed tomography revealed a highly calcified septum (white arrow) in which the residual native septum length was less than 1 cm (red arrowhead). (B) Fluoroscopic image showing a highly calcified septum (white arrow). The ablation catheter was placed onto the native atrial septum based on the 3D map and SOUND STAR image. (C, D) The voltage map showed a large scar zone (dagger) due to the septal patch (white arrow), and the surrounding native interatrial septum is shown as a low‐voltage area (asterisk).

CA was performed under the guidance of a 3D mapping system (CARTO3; Biosense Webster). A fluoroscopic image confirmed the highly calcified septum (Figure 1B). Before the TSP, a right atrial (RA) voltage map was created to assess the electrophysiological information around the atrial septum using an eight‐spline high‐resolution mapping catheter (OCTARAY™; Biosense Webster). The septal patch itself and surrounding native septum were displayed as scar and low‐voltage areas, respectively (Figure 1C,D). Intracardiac echography with a magnetic sensor (SOUND STAR; Biosense Webster), directed at this native interatrial septum revealed atrial electrical activity and depicted a small channel next to the artificial patch (Figure 2A–D; Video S1). The native septum, which we decided to target to perform the TSP, was marked on the 3D map using the ablation catheter (THERMOCOOL SMARTTOUCH™ Catheter; Biosense Webster) with a steerable sheath (Agilis NxT Steerable Introducer; Abbott), and the catheter position was recorded on the fluoroscopic image (Figures 1B and 2B). With the intracardiac echocardiography catheter held at the same position, an SL‐0 sheath was subsequently placed at the marked point (Figure 2C). The TSP was then performed with a radiofrequency needle (Radiofrequency NRG Transseptal Needle; Baylis Medical) (Figure 2E). Following the TSP, the ablation catheter passed through this puncture hole and two sheaths were successfully placed in the LA. A voltage map of the LA revealed a low‐voltage area on the posterior wall. Then, a bilateral extensive pulmonary vein isolation and linear ablation on the LA roof and inferior wall were completed (power 30–40 W, contact force 10–20 g, and target ablation index 500). A cavo‐tricuspid isthmus ablation was also performed. After the ablation, since we confirmed that atrial pacing did not induce any tachycardia including AF, the procedure was concluded. After the CA, the patient did not have any neurological disorders or decreased blood oxygen level.

**FIGURE 2:**
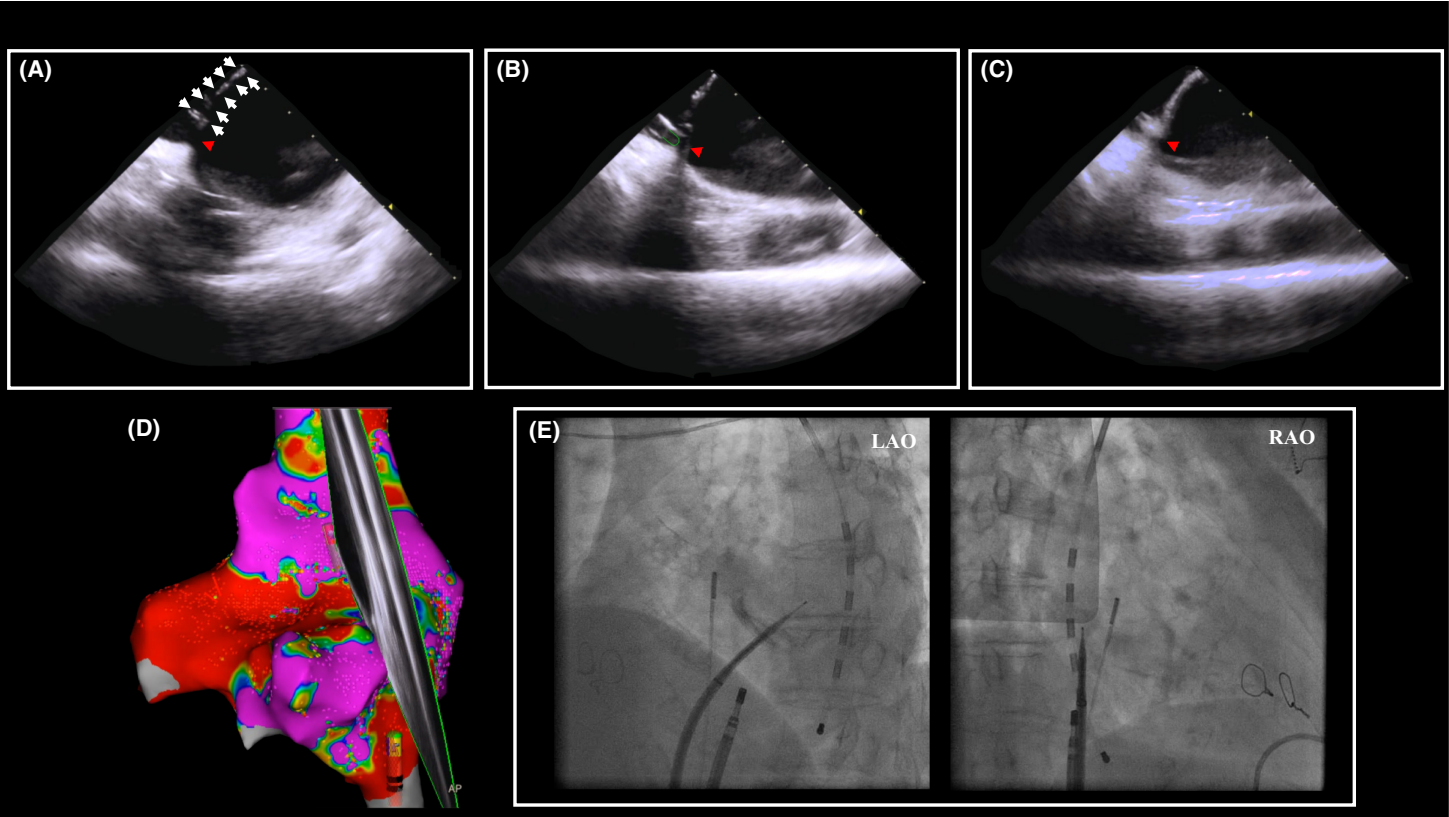
(A) Intracardiac echography revealed a small channel (arrow) with a surrounding artificial patch (arrowhead). (B) The native septum was marked using the ablation catheter based on the ultrasound image with an intracardiac magnetic sensor (arrow). (C) An SL‐0 sheath was placed on the exact location of the residual atrial septum (arrow). (D) An right atrial voltage map and the direction of the ultrasound image recorded by a SOUND STAR catheter is shown. (E) The transseptal puncture succeeded with a radiofrequency needle.

A TSP after a repaired ASD is reported to be feasible, safe, and efficacious.^2^ As discussed earlier, the TSP is performed through the repaired septum, through foreign patches, occluding devices, or the surrounding native interatrial septum.^1^ In contrast, the TSP through the repaired atrial septum in the present case was challenging due to severe calcifications. Because the calcified trans‐patch puncture precluded the possibility of a direct puncture and had a risk of an embolic event, the only viable option was to target the narrow native tissue next to the patch. The voltage map of the RA prior to the TSP was helpful to identify the precise location of the target native tissue (Figure 1C,D). Furthermore, it was critical to mark the target point on the 3D map using an ultrasound catheter with an intracardiac magnetic sensor and fluoroscopic image to keep track of the exact location of the target. A combination of these techniques critically contributed to the challenging yet successful TSP.

AF is a common arrhythmia in repaired ASD patients.^3^ It is not rare that patients develop new AF years later after the prior repair operation. However, long‐term follow up is rather rare, possibly because ASDs are classified as a simple type of congenital heart disease.^4^ Therefore, the detailed information of the prior procedure is sometimes missing. Noiri et al. reported that a preoperative imaging evaluation for a repaired ASD provided the critical information concerning the morphology of the repaired septum, even though the TTE did not reveal any abnormalities.^5^ In our case, although the prior operation information was not available and no septal calcifications were suspected on TTE, the CT images revealed severe calcifications and provided detailed information about the repaired septum (Figure 1B).

In ASD patients whose information about the prior operation is not available, a combination of a preoperative CT, RA voltage map, and intracardiac echocardiography with an intracardiac magnetic sensor would be helpful to guide a safe TSP.

## CONFLICT OF INTEREST STATEMENT

The authors declare no conflict of interest for this article.

## ETHICS STATEMENT

Not applicable.

## CONSENT FOR PUBLICATION

Patient consent for publication was obtained.

## CLINICAL TRIAL REGISTRATION

Not applicable.

## Supporting information


Video S1.
